# MDHGI: Matrix Decomposition and Heterogeneous Graph Inference for miRNA-disease association prediction

**DOI:** 10.1371/journal.pcbi.1006418

**Published:** 2018-08-24

**Authors:** Xing Chen, Jun Yin, Jia Qu, Li Huang

**Affiliations:** 1 School of Information and Control Engineering, China University of Mining and Technology, Xuzhou, China; 2 Business Analytics Centre, National University of Singapore, Singapore; University of Calgary Cumming School of Medicine, CANADA

## Abstract

Recently, a growing number of biological research and scientific experiments have demonstrated that microRNA (miRNA) affects the development of human complex diseases. Discovering miRNA-disease associations plays an increasingly vital role in devising diagnostic and therapeutic tools for diseases. However, since uncovering associations via experimental methods is expensive and time-consuming, novel and effective computational methods for association prediction are in demand. In this study, we developed a computational model of Matrix Decomposition and Heterogeneous Graph Inference for miRNA-disease association prediction (MDHGI) to discover new miRNA-disease associations by integrating the predicted association probability obtained from matrix decomposition through sparse learning method, the miRNA functional similarity, the disease semantic similarity, and the Gaussian interaction profile kernel similarity for diseases and miRNAs into a heterogeneous network. Compared with previous computational models based on heterogeneous networks, our model took full advantage of matrix decomposition before the construction of heterogeneous network, thereby improving the prediction accuracy. MDHGI obtained AUCs of 0.8945 and 0.8240 in the global and the local leave-one-out cross validation, respectively. Moreover, the AUC of 0.8794+/-0.0021 in 5-fold cross validation confirmed its stability of predictive performance. In addition, to further evaluate the model's accuracy, we applied MDHGI to four important human cancers in three different kinds of case studies. In the first type, 98% (Esophageal Neoplasms) and 98% (Lymphoma) of top 50 predicted miRNAs have been confirmed by at least one of the two databases (dbDEMC and miR2Disease) or at least one experimental literature in PubMed. In the second type of case study, what made a difference was that we removed all known associations between the miRNAs and Lung Neoplasms before implementing MDHGI on Lung Neoplasms. As a result, 100% (Lung Neoplasms) of top 50 related miRNAs have been indexed by at least one of the three databases (dbDEMC, miR2Disease and HMDD V2.0) or at least one experimental literature in PubMed. Furthermore, we also tested our prediction method on the HMDD V1.0 database to prove the applicability of MDHGI to different datasets. The results showed that 50 out of top 50 miRNAs related with the breast neoplasms were validated by at least one of the three databases (HMDD V2.0, dbDEMC, and miR2Disease) or at least one experimental literature.

## Author summary

Identifying potential miRNA-disease associations enhances the understanding towards molecular mechanisms and pathogenesis of diseases, which is beneficial for the development of diagnostic/treatment tools for diseases. Compared with traditional experiment methods, computational models can help experimenters reduce the cost of money and time. In order to computationally predict potential miRNA-disease associations, we developed MDHGI by combining the sparse learning method with the heterogeneous graph inference method. We performed MDHGI on different database and the experiment results indicated that MDHGI had significant advantages over previous methods both in leave-one-out cross validation and 5-fold cross validation. Besides, we also carried out three different kinds of case studies on four important human complex diseases to further demonstrate the prediction accuracy of MDHGI. In consequence, 98%, 98%, 100% and 100% out of the top 50 candidate miRNAs for the four diseases were confirmed by different databases or experimental literatures in PubMed, respectively. Thus, it could be concluded that MDHGI could make reliable predictions and should serve as an effective tool for predicting potential miRNA-disease associations.

## Introduction

MicroRNA (miRNA) are one class of important short noncoding RNA (~22nt) molecules that mostly inhibit gene expression at the post-transcriptional level [1–4]. In 1993, lin-4 was the first miRNA detected as a result of research on the timing of C. elegans larval development [5]. Unlike conventional protein coding genes, lin-4 coded for a 22 nucleotide regulatory RNA rather than a protein [5,6]. Since then, thousands of miRNAs have been discovered in many living organisms, and currently 2588 miRNAs in the human genome have been annotated [7]. Because each miRNA is probably able to control the expression of hundreds of target genes, the whole miRNA pathway is a critical mechanism for gene expression control [2,8–13].

Recently, more and more studies have shown that miRNAs play critical roles in diverse important biological processes. Therefore, it is no surprise that miRNA could be associated with cancers [14,15] and other kinds of diseases [16]. As indicated by increasing evidences, miRNAs are emerging as novel potential biomarkers or diagnostic/therapeutic tools for diseases [17–22]. For example, miR-708 affects the progress of bladder carcinoma through direct inhibition of Caspase-2 [23]. MiR-29c down-regulation results in derepression of its target DNA methyltransferase 3a, which promotes the development of ischemic brain damage [24]. Another example is that let-7b expression has a positive correlation with patient age (R = 0.472; p<0.001) [25]. Higher Nuclear opalescence or cataract scores for Nuclear color (N), Cortical (C) and Posterior (P) was discovered positively associated with higher expression of let-7b in patients with age-related cataracts (p<0.001) [25]. Identifying potential miRNA-disease associations enhances the understanding towards molecular mechanisms and pathogenesis of diseases. As a biomarker, miRNA can be used for disease diagnosis; and as drug targets, miRNAs can be applied to disease treatment. Since carrying out experiments is an expensive and time-consuming process, only a small number of miRNA-disease associations have been confirmed by traditional experimental approaches. Proposing computational models to predict disease-related miRNAs is a worthful supplement to experiments. Researchers should spare no effort to excogitate a more accurate prediction method so that reasonable candidates can be provided for future biological experiments [26].

In recent years, several computational methods have been developed to predict potential miRNA-disease associations and some of them performed well [27–33]. Based on the assumption that functionally related miRNAs are more likely to be associated with diseases which have similar phenotypes, Jiang *el al*. [34] proposed a network-based approach by combining miRNA similarity network, disease similarity network with miRNA-disease association network. After that, based on the hypergeometric distribution, a scoring system was constructed to acquire the scores of potential miRNA-disease associations. Focusing on the functional link between miRNA targets and disease genes, Shi *et al*. [35] devised a computational model by performing random walk on a protein-protein interaction (PPI) network and focusing on the functional links between miRNAs targets and diseases genes in PPI network. Mørk *et al*. [36] developed miRNA-Protein-Disease (miRPD) association prediction model by linking miRNAs to diseases via the underlying proteins involved. However, these methods did not exhibit a commendable predictive performance because their performance depended largely on miRNA-target interactions which have a high ratio of false-positive and false-negative samples.

Some other computational models without relying on miRNA-target interactions have been proposed in the past few years. Xuan *et al*. [37] developed the method called Human Disease-related MiRNA Prediction (HDMP) to predict miRNAs associated with diseases. The association score between a miRNA and a disease was computed by summing up the sub-scores for the miRNA’s *k* neighbors, and the sub-score for a neighbor was calculated via multiplying the weight of the neighbor with the functional similarity between the neighbor and the miRNA. However, HDMP could not be applied to new diseases without any known associated miRNAs because predictions were made mainly from the information of miRNAs’ neighbors. Based on the global similarity measures, Chen *et al*. [38] developed a method named Random Walk with Restart for MiRNA-Disease Association prediction (RWRMDA) by implementing random walk on the miRNA functional similarity network to prioritize candidate miRNAs for disease of interest. Nonetheless, this method was unable to predict miRNAs associated with the diseases without any known related miRNAs. Another computational model named MIRNAs associated with Diseases Prediction (MIDP) was developed by Xuan *et al*. [39] based on random walk on a miRNA network derived from miRNA-associated diseases and semantic similarity of their associated diseases. The model assigned higher transition weights to labeled nodes than unlabeled nodes, which efficiently exploited the prior information of nodes and the various ranges of topologies. Besides, since they extended the walking on a miRNA-disease bilayer network, MIDP could also be used to prioritize candidate miRNAs for diseases without any known associated miRNAs. Later, a method named Matrix Completion for MiRNA-Disease Association prediction (MCMDA) [40] was proposed to predict potential associations by utilizing the matrix completion algorithm to update the adjacency matrix. However, the algorithm also suffered from a limitation of not being applicable to new diseases and new miRNAs.

Recently, Chen *et al*. [41] proposed another model called Within and Between Score for MiRNA-Disease Association prediction (WBSMDA). After integrating similarity for miRNAs and diseases, within-score and between-score were calculated and combined to obtain the final score for potential miRNA-disease association inference. Later, Chen *et al*. [42] presented a model of Heterogeneous Graph Inference for MiRNA-Disease Association prediction (HGIMDA) by combining the integrated miRNA similarity network, the integrated disease similarities network and the known miRNA-disease associations network into a heterogeneous graph. After that, they constructed an iterative equation by summarizing all paths with the length equal to three from which they can infer potential association between a disease and a miRNA.

In addition, several other computational models were based on machine learning algorithms. For example, based on the features which were extracted from MiRNA Target-Dysregulated Network (MTDN) model by assessing topological properties of miRNAs and changes in miRNA expression, Xu *et al*. [43] implemented a Support Vector Machine (SVM) classifier to distinguish positive miRNA-disease associations from negative ones. However, even till today it is still difficult to obtain negative samples, and this fact seriously decreased the prediction accuracy of MTDN. Chen *et al*. [44] further presented Regularized Least Squares for MiRNA-Disease Association prediction (RLSMDA) method based on semi-supervised learning in the miRNA space and the disease space. What is worth mentioning is that RLSMDA could identify related miRNAs for diseases without any known associated miRNAs. Chen *et al*. [45] developed another computational model called restricted Boltzmann machine for multiple types of miRNA-disease association prediction (RBMMMDA), the core of which was restricted Boltzmann machine (RBM). The model built a two-layer undirected graphical model containing layers of visible and hidden units. Compared to previous models, RBMMMDA could obtain not only new miRNA-disease associations but also the corresponding association types. The method named Ranking-based KNN for miRNA-Disease Association prediction (RKNNMDA) [46] was first implemented to search for *k*-nearest neighbors both for miRNAs and diseases by using the *K*-Nearest Neighbors (KNN) algorithm. Then these *k*-nearest neighbors were reranked according to the SVM ranking model. Finally, weighted voting was carried out on the ranking results to obtain the final ranking of all potential miRNA-disease associations. The drawback of RKNNMDA was that bias might be caused to miRNAs with more known associated diseases.

Identifying miRNAs associated with diseases is beneficial for the development of diagnostic/treatment tools for diseases. Using traditional experimental methods for association detection is demanding and so computational models for miRNA-disease association prediction are needed to complement to experiments. Because previously developed computational methods have some aforementioned limitations, it is essential to develop a new method that exploits more useful information and make more reliable predictions. However, there are also some difficulties of predicting potential disease-related miRNAs, such as the rare known miRNA-disease associations, the unavailable negative miRNA-disease associations, the relatively limited biological datasets about miRNAs, and the universality to new diseases without any known associated miRNAs as well as new miRNAs. What’s more, considering that some of the existing computational models are only based on one of the matrix decomposition algorithm and network algorithm, it is of great significance to fully take advantage of these two methods to develop a new calculation model for miRNA-disease association prediction. In this study, we developed an effective computational model of Matrix Decomposition and Heterogeneous Graph Inference for miRNA-disease association prediction (MDHGI). We first rebuilt a new adjacency matrix by using Sparse Learning Method (SLM) to decompose the original adjacency matrix obtained from known miRNA-disease associations. Then we combined the miRNA functional similarities network, the disease semantic similarities network, the Gaussian interaction profile kernel similarities network, and the new adjacency matrix into a heterogeneous graph. Finally, we implemented normalization on integrated similarity for miRNAs and diseases and iteration algorithm on the graph to obtain a predicted association score scores for all miRNA-disease pairs. To evaluate the effectiveness of MDHGI, global and local Leave-One-Out Cross Validation (LOOCV) as well as 5-fold cross validation were carried out. The AUCs of global and local LOOCV were respectively 0.8945 and 0.8240, and the model obtained an average AUC of 0.8794+/-0.0021 in 5-fold cross validation. In the case studies of four important human cancers, 49, 49, 50, and 50 out top 50 predicted miRNAs for Esophageal Neoplasms, Lymphoma, Lung Neoplasms, and Breast Neoplasms were respectively confirmed by different databases or experimental literatures in PubMed. These results proved that MDHGI was effective in predicting potential miRNA-disease associations and it had significant advantages over previous methods. Our main contribution in this article is to perfect the HGIMDA model and further improve its accuracy by taking full advantage of the two methods (matrix decomposition and network algorithm). Besides, the idea presented in this article may have new inspiration for other researchers and the model we proposed is also a supplement to methodological research.

## Materials and methods

### Human miRNA-disease associations

Actually, since the data in the databases is derived from the collected experimental literatures, it is a common practice for researchers to utilize known miRNA-disease associations data in HMDD as the training set. As previous studies have done [27,39,47–49], in this paper, the known miRNA-disease associations dataset was extracted from the HMDD V2.0 database. The dataset contained 5430 validated associations between 495 miRNAs and 383 diseases. To facilitate subsequent calculations, we constructed an adjacency matrix *A* ∈ *R*^*m*×*n*^ to store the known miRNA-disease associations and other miRNA-disease pairs. In the adjacency matrix *A*, *m* and *n* are respectively defined as the number of miRNAs and diseases. Besides, element *A*(*r*_*i*_,*d*_*j*_) is set to be 1 if miRNA *r*_*i*_ is associated with disease *d*_*j*_, otherwise 0 [42].

$$A_{ij}=\left\{ \begin{array}{ll} 1, & \mathrm{if}\;\mathrm{miRNA}\;r_{i}\;\mathrm{associated}\;\mathrm{with}\;\mathrm{disease}\;d_{j}\\ 0, & \mathrm{otherwise} \end{array}\right.$$ (1)

### Disease semantic similarity model 1

**Fig 1:**
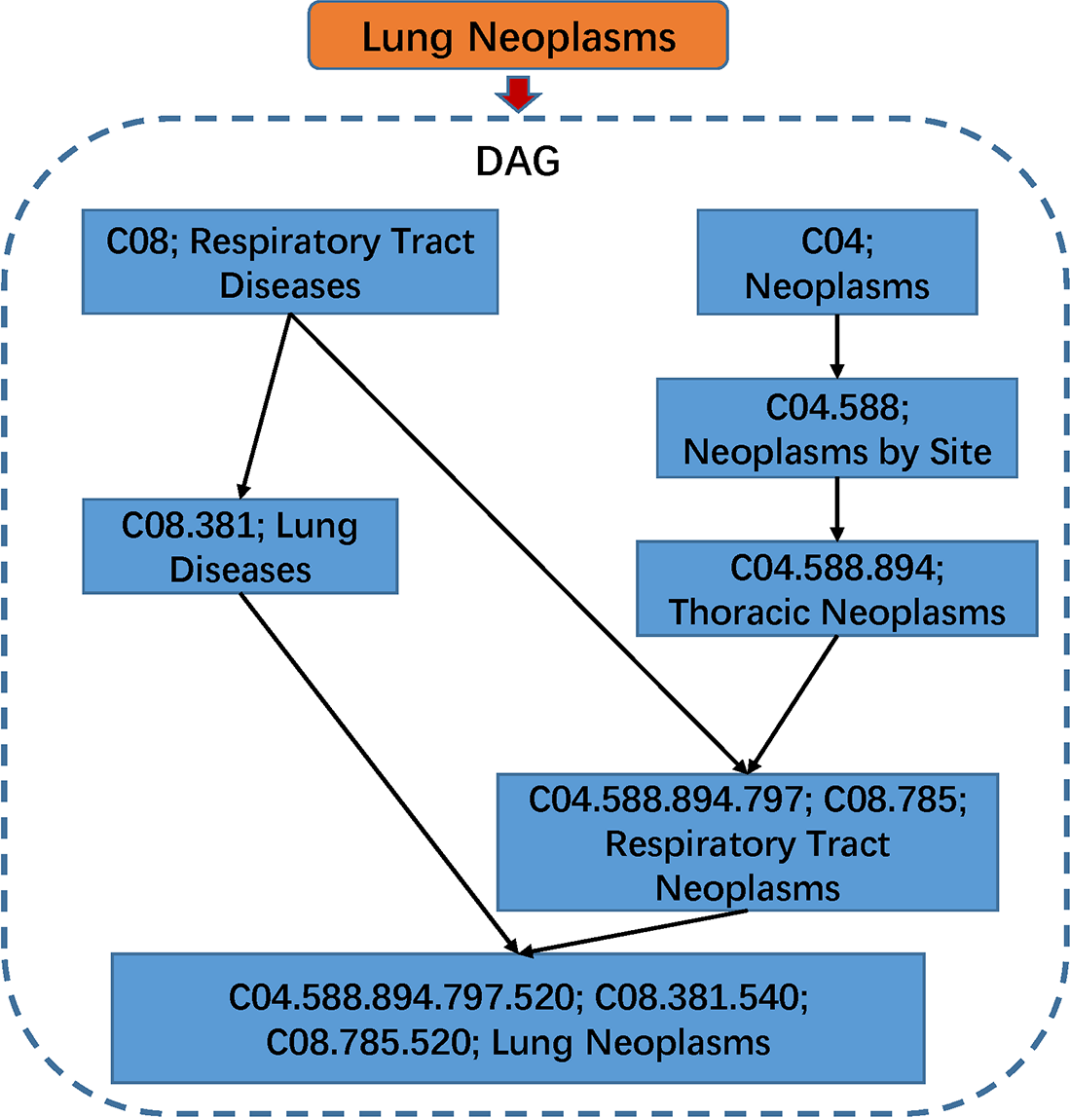
The disease DAG of lung neoplasms. The addresses of its ancestors are shown in a DAG structure.

In previous studies [50–54], many researchers made use of the DAG to describe a disease in their calculation models. According to the National Library of Medicine (http://www.nlm.nih.gov/), we can obtain the relationship of various diseases based on the disease Directed Acyclic Graph (DAG) constructed from the MeSH descriptor of Category C. For example, for the DAG of lung neoplasms (See Fig 1), ‘respiratory tract diseases’ points to ‘lung diseases’. All nodes in the DAG are connected by a direct edge from a more general term, we call it parent, to a more specific term, and we call it child [55]. Here, a disease *D* was described by *DAG* = (*D*,*T*(*D*),*E*(*D*)), in which we defined all ancestor nodes of *D* and *D* itself as *T*(*D*) and the edge set including the direct edges from parent nodes to child nodes as *E*(*D*). In DAG(*D*), the contribution of disease *d* to the semantic value of disease *D* was defined as:

$$\left\{ \begin{array}{l} D1_{D}\left(d\right)=1\;if\;d=D\\ D1_{D}\left(d\right)=\max\;\left\{\Delta *D1_{D}\left(d^{\prime }\right)|d^{\prime }\in children\;of\;d\right\}\;if\;d\neq D \end{array}\right.$$ (2)

where Δ is the semantic contribution decay factor. Here, based on the previous literature [37], we denoted the value of Δ to 0.5. Moreover, the semantic value of disease *D* was defined as:

$$DV1\left(D\right)=\sum_{d\in T(D)}D1_{D}(d)$$ (3)

Since the larger part of DAG was shared by two diseases, the higher semantic similarity value they would get, the semantic similarity score between disease *d*_*i*_ and *d*_*j*_ were defined as follows:

$$SS1(d_{i},d_{j})=\frac{\sum_{t\in T(d_{i})\cap T(d_{j})}\left(D1_{d_{i}}(t)+D1_{d_{j}}(t)\right)}{DV1(d_{i})+DV1(d_{j})}$$ (4)

### Disease semantic similarity model 2

It is obvious that a disease which appears in less DAGs contributes to the semantic similarity of disease at a high level. Considering the inexact approach in disease semantic similarity model 1 that the contribution of diseases in the same layer of *DAG* (*D*) to the semantic value of *D* were treated as the same. We defined the contribution of disease *d* in DAG(*D*) to the semantic value of disease *D* as follows:

$$D2_{D}(d)=-\log[\mathrm{the}\;\mathrm{number}\;\mathrm{of}\;\mathrm{DAGs}\;\mathrm{including}\;t/\mathrm{the}\;\mathrm{number}\;\mathrm{of}\;\mathrm{diseases}]$$ (5)

We then defined the semantic similarity between disease *d*_*i*_ and *d*_*j*_ in the similar way as the disease semantic similarity model 1.

$$SS2(d_{i},d_{j})=\frac{\sum_{t\in T(d_{i})\cap T(d_{j})}\left(D2_{d_{i}}(t)+D2_{d_{j}}(t)\right)}{DV2(d_{i})+DV2(d_{j})}$$ (6)

$$DV2\left(D\right)=\sum_{d\in T(D)}D2_{D}(d)$$ (7)

### MiRNA functional similarity

Wang *et al*. [56] developed the MISIM method to calculate the miRNA functional similarity between a miRNA pair (*r*_*i*_ and *r*_*j*_). The whole process of MISIM can be divided into four steps. In the first step, we need to identify the diseases set *D*(*r*_*i*_) (diseases associated with *r*_*i*_) and *D*(*r*_*j*_) (diseases associated with *r*_*j*_) for miRNA *r*_*i*_ and *r*_*j*_, respectively. Next, the semantic value of all diseases in these two sets are computed according to the corresponding DAG. Third, the semantic similarity for each disease pairs between *D*(*r*_*i*_) and *D*(*r*_*j*_) can be calculated based on their semantic value. Finally, the functional similarity of *r*_*i*_ and *r*_*j*_ is calculated based on the semantic similarity obtained in step three. From the website http://www.cuilab.cn/files/images/cuilab/misim.zip, we downloaded the miRNA functional similarity data. Then the miRNA functional similarity matrix *MS* was constructed, in which the element *MS*(*r*_*i*_,*r*_*j*_) indicated the similarity value between the miRNA *r*_*i*_ and the miRNA *r*_*j*_.

### Gaussian interaction profile kernel similarity

Based on the notion that functionally similar miRNAs are usually associated with similar diseases, the Gaussian interaction profile kernel similarity can be constructed as another algorithm for similarity measurement between two miRNAs/diseases [57,58]. It is obvious that the *i*^*th*^ row and *j*^*th*^ column of adjacent matrix *A* respectively represents the information whether the miRNA or the disease are associated with each of the diseases or the miRNAs. For convenience, we denoted vector *IV*(*r*_*i*_) and *IV*(*d*_*j*_) to represent the *i*^*th*^ row vector and *j*^*th*^ column vector, respectively. Therefore, the Gaussian interaction profile kernel similarity of diseases and miRNAs could be computed as follows:

$$GD(d_{i},d_{j})=\exp(-\beta_{d}{\left\|IV(d_{i})-IV(d_{j})\right\|}^{2})$$ (8)

$$GR(r_{i},r_{j})=\exp(-\beta_{r}{\left\|IV(r_{i})-IV(r_{j})\right\|}^{2})$$ (9)

where the adjustment coefficients *β*_*d*_ and *β*_*r*_ could be defined as follows:

$$\beta_{d}=\beta {'}_{d}/\left(\frac{1}{n}\sum_{i=1}^{n}{\left\|IV(d_{i})\right\|}^{2}\right)$$ (10)

$$\beta_{r}=\beta {'}_{r}/\left(\frac{1}{m}\sum_{i=1}^{m}{\left\|IV(r_{i})\right\|}^{2}\right)$$ (11)

where *β*′_*d*_ and *β*′_*r*_ are the original bandwidths and both of them were defined as 1 based on the previous study [59].

### Integrated similarity for miRNAs and diseases

The integrated disease similarity could be obtained through combining the disease semantic similarity and the disease Gaussian interaction profile kernel similarity. What makes a difference to the integrated miRNA similarity is that if disease *d*_*i*_ and *d*_*j*_ have their own DAG (i.e. these two diseases have semantic similarity), then the final integrated similarity is the average between *SS* and *GD*. Otherwise the integrated disease similarity equals to the value of Gaussian interaction profile kernel similarity.

$$SS(d_{i},d_{j})=\frac{SS1(d_{i},d_{j})+SS2(d_{i},d_{j})}{2}$$ (12)

$$SD(d_{i},d_{j})=\left\{ \begin{array}{l} \frac{\left(\mathrm{SS}(d_{i},d_{j})+GD(d_{i},d_{j})\right)}{2}\;d_{i}\;\mathrm{and}\;d_{j}\;\mathrm{have}\;\mathrm{their}\;\mathrm{own}\;\mathrm{DAG}\\ GD(d_{i},d_{j})\;\mathrm{otherwise} \end{array}\right.$$ (13)

Furthermore, the integrated miRNA similarity could be obtained by combining the miRNA functional similarity with miRNA Gaussian interaction profile kernel similarity:

$$SR(r_{i},r_{j})=\left\{ \begin{array}{l} \frac{\left(MS(r_{i},r_{j})+GR(r_{i},r_{j})\right)}{2}\;r_{i}\;\mathrm{and}\;r_{j}\;\mathrm{has}\;\mathrm{functional}\;\mathrm{similarity}\\ GR(r_{i},r_{j})\;\mathrm{otherwise} \end{array}\right.$$ (14)

### MDHGI

**Fig 2:**
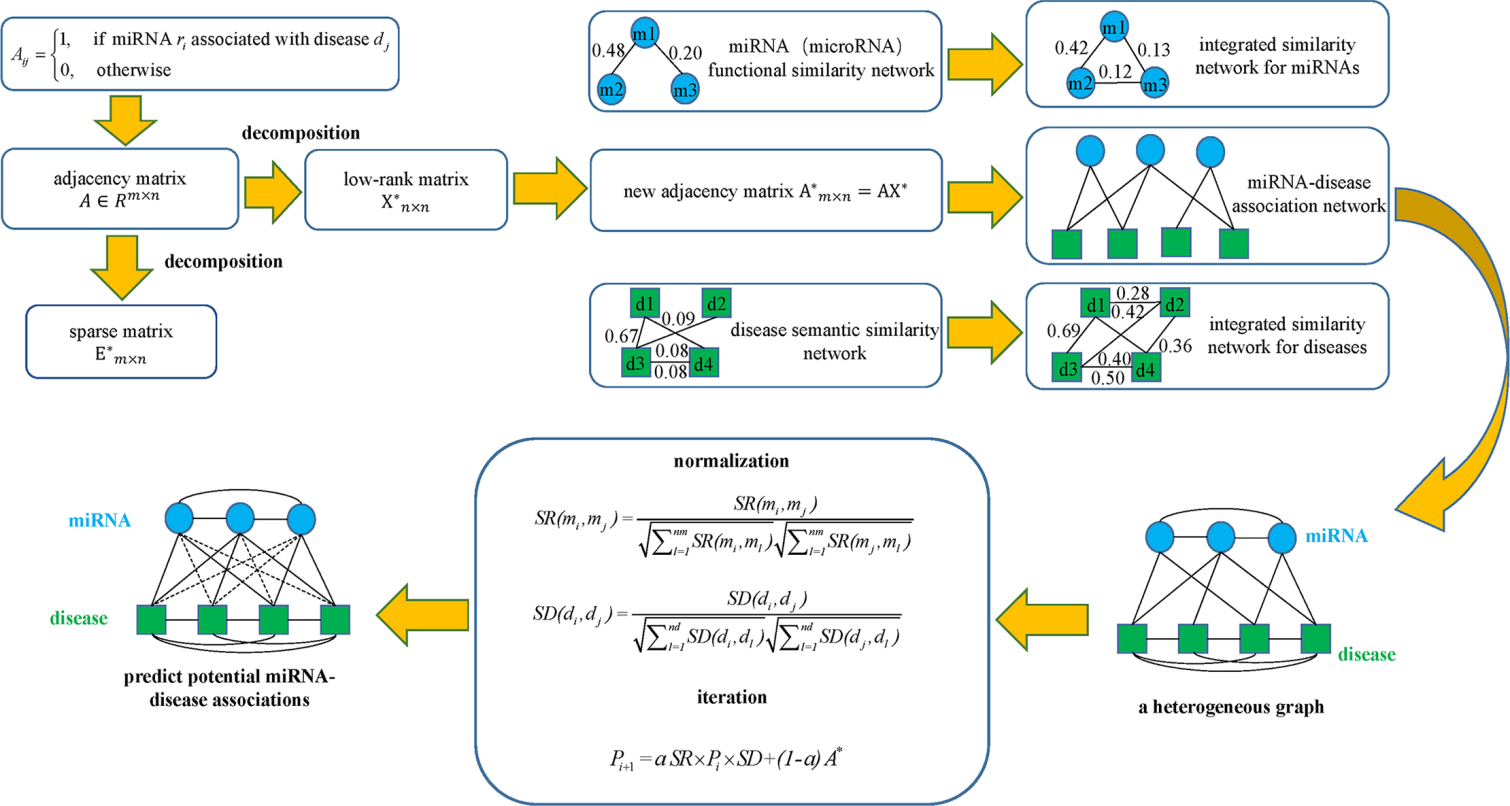
Flowchart of MDHGI model to predict the potential miRNA-disease associations based on the known associations in HMDD V2.0 database.

In this study, the proposed method, MDHGI, fully extends the advantages of matrix factorization and network algorithm to make prediction for miRNA-disease associations. The flow chart of the algorithm is shown in Fig 2.

Actually, the data we used to train our model are normally far from perfect. Considering that, a portion of the miRNA-disease associations in the real data would be redundant, and also some other miRNA-disease associations would be missing from the real data. Hence, the adjacency matrix for miRNA-disease associations can be decomposed into two parts. The first part is a linear combination of the original adjacency matrix and a low-rank matrix and the second part is a sparse matrix with most entries being zeros and can be considered as the noise or the outliers. The method is used to look for the lowest-rank matrix which is further utilized to reconstruct a new adjacency matrix that will be used in the next calculation.

Firstly, we decomposed *A* as follows:

A=AX+E (15)

Obviously, there were infinite many solutions for Eq (15). However, since we wished *X* to be of low rank, where rank of a matrix was the maximum number of linearly independent column (or row) vectors in the matrix, and *E* to be sparse, we could enforce the nuclear norm or trace norm on *X* and sparse norm on *E*. Mathematically, Eq (15) could be thus relaxed as

$$\underset{X,E}{min}{\left\|X\right\|}_{*}+\alpha {\left\|E\right\|}_{2,1}\;s.t.\;A=AX+E$$ (16)

where

$${\left\|X\right\|}_{*}=\sum_{i}\sigma_{i}(i.e.,\sigma_{i}\;\mathrm{is}\;\mathrm{the}\;\mathrm{sigular}\;\mathrm{values}\;\mathrm{of}\;X)$$ (17)

$${\left\|E\right\|}_{2,1}=\sum_{j=1}^{n}\sqrt{\sum_{i=1}^{n}{\left(E_{ij}\right)}^{2}}$$ (18)

*α* is a positive free parameter which was used to balance the weights of low-rank matrix and sparse matrix. Here, according to the existing method [60], the value of *α* was defined as 0.1. Minimizing the trace norm of a matrix contributed to the lower-rank matrix, meanwhile the sparse norm was capable of identifying noises and outliers.

If the matrix *A* in *AX* in the right side of Eq (16) is set as identity matrix, then the model is degenerated to the robust PCA. Therefore, Eq (16) could also be regarded as a generalization of the robust PCA [61,62]. Eq (16) could be rewritten into an equivalent problem as

$$\underset{X,E,J}{min}{\left\|J\right\|}_{*}+\alpha {\left\|E\right\|}_{2,1}\;s.t.\;A=AX+E,X=J$$ (19)

**Fig 3:**
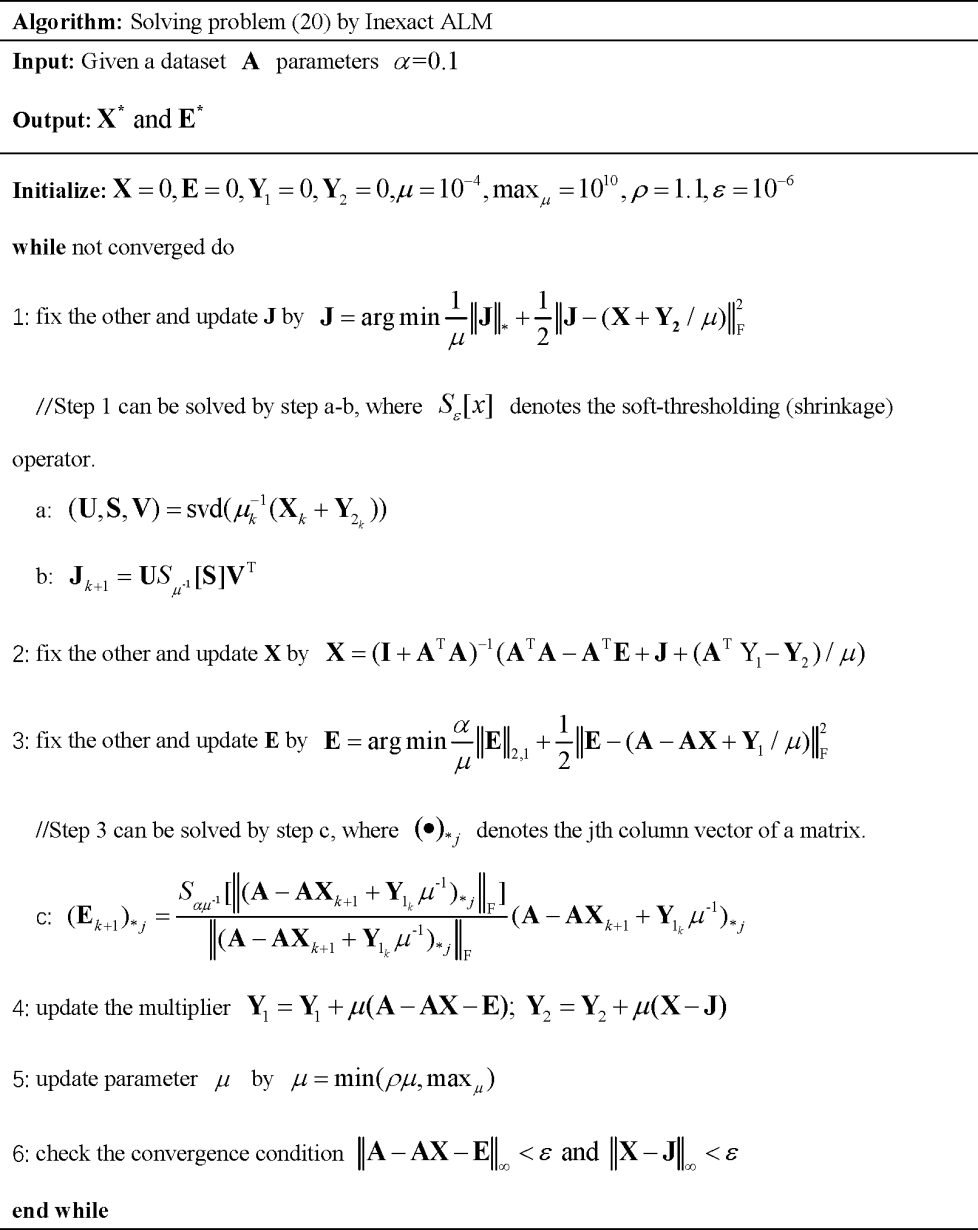
The illustration of the inexact ALM algorithm.

The Eq (19) above, which is a constraint and convex optimization problem, can be solved by off-the-shelf interior point solvers after being reformulated as a semidefinite program [63]. However, the interior point solvers are not suitable for large matrices since they rely much on second-order information of the objective function. Thus, we should take advantage of both the first-order information and the special properties of this class of convex optimization problems to overcome the scalability issue. The iterative thresholding (IT) algorithm, accelerated proximal gradient (APG) algorithm, exact augmented Lagrange multipliers (EALM) algorithm and inexact augmented Lagrange multipliers (IALM) algorithm are several methods to solve the problem of Eq (19). However, for IT algorithm, as shown in the original literature [55], the iteration process converges extremely slowly (about 10^4^ iterations to converge). As for APG algorithm, although the APG's computing speed has improved when compared to IT algorithm, it is still not as fast as IALM. Especially, the solution to Eq (19) obtained from the IALM is much more accurate than that by APG. Moreover, even though the convergence rate of EALM is as fast as IALM, the latter requires less number of partial SVDs. In general, the IALM algorithm is a relatively more efficient algorithm to solve the problem of Eq (19). Thus, in this paper, we utilized IALM [64] method by first converting Eq (19) to an unconstraint problem and then minimizing this problem based on augmented Lagrange function such that

$$L={\left\|J\right\|}_{*}+\alpha {\left\|E\right\|}_{2,1}+tr(Y_{1}^{T}(A‑AX‑E))+tr(Y_{2}^{T}(X‑J))+\frac{\mu }{2}({\left\|A‑AX‑E\right\|}_{F}^{2}+{\left\|X‑J\right\|}_{F}^{2})$$ (20)

where *μ* ≥ 0 is a penalty parameter. The problem above could be solved by minimizing with respect to *J*, *X*, and *E*, respectively. Besides, after fixing the other variables and then updating the Lagrange multipliers *Y*_1_, *Y*_2_, Eq (20)would be settled. The detailed steps of how to solve Eq (20) is shown in Fig 3.

We defined the solution of Eq (20) as X* and E*. If *A*_*ij*_ represents the association between miRNA *r*_*i*_ and disease *d*_*j*_, then X* ∈ *R*^*n*×*n*^ could be considered as a similarity matrix that described the similarity between diseases. While if *A*_*ij*_ represents the associations between disease *d*_*i*_ and miRNA *r*_*j*_ (as the transposition of the adjacency matrix in Eq (15)), then X* ∈ *R*^*m*×*m*^ describes the similarity between miRNAs. After obtaining X*, the solution of Eq (20), we could compute the new associations between each pair of miRNAs and diseases by projecting the adjacency matrix onto the lower-dimensional space as

$$A^{*}=AX^{*}$$ (21)

From the matrix A*, we reacquired the miRNA-disease associations information which were further combined with the integrated similarity for miRNAs and diseases into a heterogeneous graph. By analyzing the heterogeneous graph, for disease *d* and miRNA *m*, we could further define their potential association probability as follows if they had no known associations.

$$P(m,d)=\sum_{i=1}^{nm}\sum_{j=1}^{nd}SR(m_{i},m)*A^{*}(m_{i},d_{j})*SD(d_{j},d)$$ (22)

**Fig 4:**
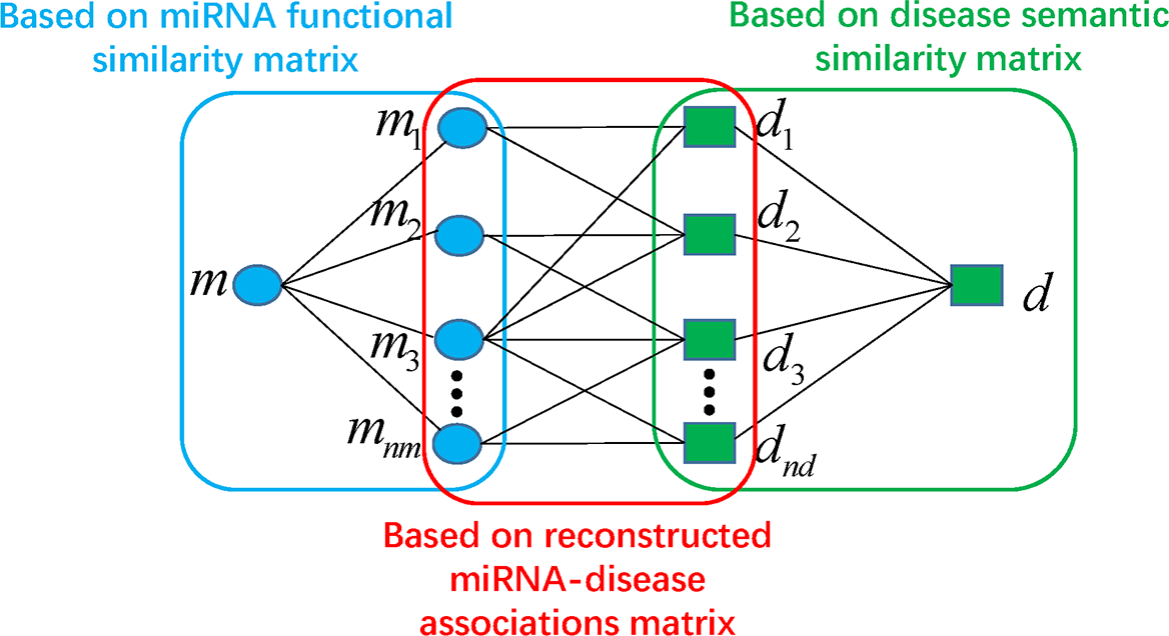
The potential association probability between the miRNA *m* and the disease *d* which can be calculated by summarizing all paths with the length equal to three (For example, *m*–*m*_1_–*d*_1_–*d*).

According to the equation above, the potential association probability between miRNA *m* and disease *d* could be calculated by summarizing all paths with the length equal to three (See Fig 4). Moreover, considering the iteration of above process, we obtained iterative equation through representing the equation as matrix multiplications.

$$P_{i+1}=a\;SR\times P_{i}\times SD+(1‑a)\;A^{*}$$ (23)

Here, the decay factor *a* was denoted to 0.4 based on the previous study [65]. For the iteration, we can treat this process like that every node with prior information disseminates the information obtained in the previous iteration to its neighbors. Due to the relation between the end-points and the probability of looking into an edge among the same end-points in a random network with the same node degrees, the weight of an edge was normalized according to the degrees of its end-points [66]. Based on the previous literature [65], miRNA-disease association probability matrix *P* would converge when *SR* and *SD* were properly normalized utilizing Eqs (24) and (25), respectively. Moreover, we have given the specific proof process as Theorem 1 in the S1 Text

$$SR(m_{i},m_{j})=\frac{SR(m_{i},m_{j})}{\sqrt{\sum_{l=1}^{nm}SR(m_{i},m_{l})}\sqrt{\sum_{l=1}^{nm}SR(m_{j},m_{l})}}$$ (24)

$$SD(d_{i},d_{j})=\frac{SD(d_{i},d_{j})}{\sqrt{\sum_{l=1}^{nd}SD(d_{i},d_{l})}\sqrt{\sum_{l=1}^{nd}SD(d_{j},d_{l})}}$$ (25)

Here, we set the cutoff as 10^−6^. The iteration above would become stable when the change between *P*_*i*_ and *P*_*i*+1_ measured by L1 norm was less than the given cutoff.

In addition, for convenience, we have made a web server at http://chengroup.cumt.edu.cn/tool/mdhgi/. After opening the website and selecting the disease name of interest in the box, researchers will get the prediction results of potential disease-related miRNAs. For more details, please see the website's ‘Guide’.

## Results

### Performance evaluation

Here, we used two types of cross validation to evaluate the performance of MDHGI, namely, LOOCV and 5-fold cross validation. LOOCV could be further divided into global and local LOOCV, in which each known association was in turn considered to be the test sample and the others were treated as the training samples. In global LOOCV, each of the known miRNA-disease associations was in turn considered as the test sample and all unknown miRNA-disease pairs were treated as candidate samples, while in local LOOCV, candidate samples only contained those miRNAs without any known associations with the investigated disease in the test sample. In 5-fold cross validation, we randomly divided all known miRNA-disease associations into five subsets with equal size. Then each subset was in turn considered as the test sample and the rest four subsets were treated as training samples. In the same way as LOOCV, all unknown miRNA-disease pairs were regarded as candidate samples. Subsequently, we obtained a predicted association score matrix by MDHGI, and ranked the score of each test sample against the scores of the candidate samples. This partition-prediction-ranking procedure was repeated 100 times to obtain a sound estimate of the mean and variance of MDHGI’s prediction accuracy.

**Fig 5:**
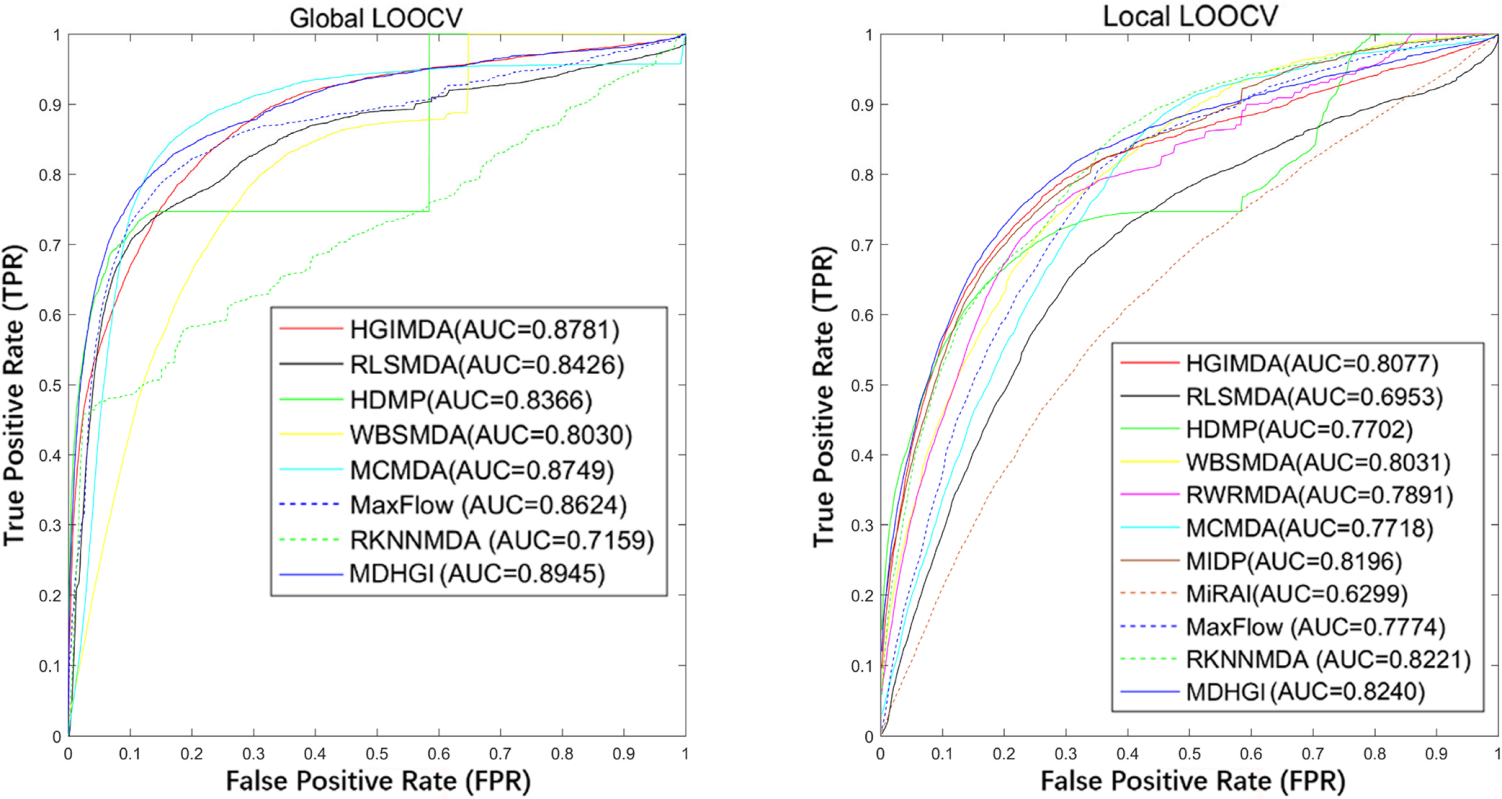
The left graph shows the AUC of global LOOCV compared with HGIMDA, RLSMDA, HDMP, WBSMDA, and MCMDA. The right graph shows the AUC of local LOOCV compared with HGIMDA, RLSMDA, HDMP, WBSMDA, MCMDA, RWRMDA, MIDP, and MiRAI. As a result, MDHGI achieved AUCs of 0.8945 and 0.8240 in the global and local LOOCV, which exceed all the previous classical models.

In each cross validation scheme, the model would be considered to successfully predict an association if the ranking of a test sample was above a given threshold. Moreover, we drew a receiver operating characteristics (ROC) curve through plotting the true positive rate (TPR, sensitivity) versus the false positive rate (FPR, 1-specificity) at different thresholds. Sensitivity referred to as the percentage of the test samples whose ranks surpassed the given threshold, while specificity denoted the percentage of negative miRNA-disease associations whose ranks were below the threshold. Then, we calculated the area under the ROC curve (AUC) to evaluate the predictive performance of MDHGI. AUC = 1 would indicate that all test samples were perfectly predicted, while AUC = 0.5 would mean the model only had random prediction performance. As shown in Fig 5, MDHGI obtained an AUC of 0.8945 in global LOOCV and an AUC of 0.8240 in local LOOCV. These results proved that MDHGI exhibited a sound performance in predicting potential miRNA–disease associations. However, the AUCs for MaxFlow [67], RKNNMDA [46], HGIMDA [42], RLSMDA [44], HDMP [68], WBSMDA [41], and MCMDA [40] in global LOOCV were 0.8624, 0.7159, 0.8781, 0.8426, 0.8366, 0.8030, and 0.8749, respectively. In local LOOCV, these models’ AUCs were 0.7774, 0.8221, 0.8077, 0.6953, 0.7702, 0.8031, and 0.7718, respectively. In addition, the AUCs in local LOOCV for RWRMDA [38], MIDP [39] and MiRAI [69] were 0.7891, 0.8196 and 0.6299, respectively. Both RWRMDA and MIDP were not applicable to global LOOCV, because, based on random walk, they could not uncover missing associations for all the diseases simultaneously. Moreover, MiRAI was also not included in global LOOCV. In MiRAI, for a disease/miRNA associated with more miRNAs/diseases, the association scores between the disease/miRNA and its candidate miRNAs/diseases tended to be higher. Therefore, the association scores obtained for different diseases were not comparable. MiRAI had a low AUC because our training dataset was sparse. Since the dataset only contained 5430 validated associations between 495 miRNAs and 383 diseases, the majority miRNAs/diseases were associated with only a few diseases/miRNAs. While in the original literature [69], the dataset contained only 83 diseases with at least 20 known associated miRNAs. As for 5-fold cross validation, in comparison with MaxFlow, RKNNMDA, RLSMDA, HDMP, WBSMDA and MCMDA whose average AUCs were 0.8579+/-0.001, 0.6723+/-0.0027, 0.8569+/-0.0020, 0.8342+/-0.0010, 0.8185+/-0.0009 and 0.8767+/-0.0011, respectively, the average AUC for MDHGI was 0.8794+/-0.0021. This further confirmed the superior prediction accuracy and the performance stability of our model.

In addition, we have supplemented seven experiments by assigning different weight parameters to miRNA-miRNA edges and disease-disease edges, while the weight for miRNA-disease edges remains unchanged (See Table 1). The reason why we carried out these experiments is that we can make quantitative analysis for the reliability of the data (the miRNA functional similarity and the disease semantic similarity). As shown in Table 1, with the diminution of the weight for miRNA-miRNA edge and disease-disease edge, the values of the AUC for Global LOOCV, Local LOOCV and 5-fold cross validation decreased. What is worth mentioning is that all of the three types of AUCs descended in a very slow way, which proved our model’s stability in a certain degree. From the results, we can conclude that the data of miRNA functional similarity and disease semantic similarity we used are reliable.

**Table 1:** Supplementary experiments with different weight parameters to miRNA-miRNA edges and disease-disease edges (bold fonts are original weights and results).

The weight for miRNA-miRNA edge	The weight for disease-disease edge	The weight for miRNA-disease edge	The AUC for Global LOOCV	The AUC for Local LOOCV	The AUC for 5-fold cross validation
**1**	**1**	**1**	**0.8945**	**0.8240**	**0.8794+/-0.0021**
0.9	0.9	1	0.8925	0.8226	0.8774+/-0.0019
0.8	0.8	1	0.8903	0.8214	0.8751+/-0.0021
0.7	0.7	1	0.8881	0.8201	0.8724+/-0.0021
0.6	0.6	1	0.8859	0.8190	0.8708+/-0.0021
0.5	0.5	1	0.8839	0.8180	0.8681+/-0.0019
0.4	0.4	1	0.8822	0.8172	0.8666+/-0.0017
0.3	0.3	1	0.8807	0.8165	0.8650+/-0.0022

### Case studies

In order to further demonstrate the prediction accuracy of MDHGI, we carried out case studies on two important human complex diseases by prioritizing candidate miRNAs for the diseases using our model with the training dataset from HMDD V2.0 [70]. Just like the validation databases in some existing methods [27,39,47–49], we verified the top 50 predictions with two other prominent miRNA-disease association databases, namely, dbDEMC [71] and miR2Disease [72]. The first type of case study was implemented on Esophageal Neoplasms and Lymphoma. In our model, we utilized the known miRNA-disease associations in HMDD V2.0 as the training set. After ranking all candidate miRNAs for each investigated disease based on their predicted scores, the top 50 predicted miRNAs were picked out and verified in other two prominent miRNA-disease association databases (i.e., dbDEMC and miR2Disease). Besides, the results showed that 232 of the 5430 known miRNA-disease associations in HMDD V2.0 also existed in miR2Disease and 546 associations also existed in dbDEMC after comparing the HMDD V2.0 with miR2Disease/dbDEMC. Nonetheless, since only candidate miRNAs (miRNAs unassociated with the investigated disease based on HMDD V2.0) for an investigated disease were ranked and verified, there was no overlap between the training samples and the prediction lists. Hence, none of the top 50 predicted miRNAs existed in HMDD V2.0 and the verification of miRNAs in the prediction lists was completely independent of HMDD V2.0.

Esophageal cancer is a commonly-diagnosed cancer arising from the esophagus—the food pipe that runs between the throat and the stomach. Based on the estimates of the esophageal cancer burden in the United States in 2017, the new cases and deaths from esophageal cancer will be 16940 and 15690, respectively [73]. Recent research showed that the first miRNA we predicted (hsa-mir-200b) suppresses invasiveness and modulates the cytoskeletal and adhesive machinery in esophageal squamous cell carcinoma cells via targeting Kindlin-2 [74]. Moreover, the data provided by Chen *et al*. [75] offered the convincing evidence that combined expression of hsa-mir-133a and hsa-mir-133b (2nd in the prediction list) might predict chemosensitivity of patients with esophageal squamous cell carcinoma (ESCC) undergoing paclitaxel-based chemotherapy which implied its importance in applying ‘personalized cancer medicine’ in the clinical treatment of ESCC. Another example is that aberrant expression level of hsa-mir-16 (3rd in the prediction list) could suppress cell apoptosis while promote growth by regulating the reversion-inducing cysteine-rich protein with Kazal motifs (RECK) and the ex-determining region Y-related high-mobility-group box transcription factor 6 (SOX6) which play important roles in the pathogenesis of ESCC [76]. MDHGI was implemented to identify potentially related miRNAs for Esophageal Neoplasms and ranked the miRNAs in terms of their association scores. As a result, 10 out of the top 10, 18 out of the top 20, and 43 out of the top 50 predictions were manually confirmed in database dbDEMC and miR2disease (See Table 2). Besides, to further confirmed our prediction results, we also manually verified the top 50 predicted miRNAs in PubMed. The result showed that 49, 49 and 46 out of the top 50 predictions were respectively confirmed by at least one, two, and three experimental literatures in PubMed (See S1 Table).

**Table 2:** Prediction of the top 50 predicted miRNAs associated with Esophageal Neoplasms based on known associations in HMDD V2.0 database. The prediction result was examined in dbDEMC and miR2Disease. The first column records top 1–25 related miRNAs. The third column records the top 26–50 related miRNAs.

miRNA	Evidence	miRNA	Evidence
hsa-mir-200b	dbDEMC	hsa-mir-10a	dbDEMC
hsa-mir-133b	dbDEMC	hsa-mir-182	dbDEMC
hsa-mir-16	dbDEMC	hsa-mir-127	dbDEMC
hsa-mir-19b	dbDEMC	hsa-mir-320a	unconfirmed
hsa-mir-429	dbDEMC	hsa-mir-193b	dbDEMC
hsa-mir-17	dbDEMC	hsa-mir-27b	dbDEMC
hsa-mir-125b	dbDEMC	hsa-mir-181b	dbDEMC
hsa-mir-142	dbDEMC	hsa-mir-29a	dbDEMC
hsa-mir-1	dbDEMC	hsa-mir-7	dbDEMC
hsa-mir-199b	dbDEMC	hsa-mir-191	dbDEMC
hsa-let-7d	dbDEMC	hsa-let-7f	unconfirmed
hsa-mir-218	unconfirmed	hsa-mir-124	dbDEMC
hsa-mir-195	dbDEMC	hsa-mir-378a	unconfirmed
hsa-mir-708	unconfirmed	hsa-mir-125a	dbDEMC
hsa-mir-10b	dbDEMC	hsa-mir-222	dbDEMC
hsa-mir-30c	dbDEMC	hsa-mir-15b	dbDEMC
hsa-mir-194	dbDEMC;miR2Disease	hsa-mir-197	dbDEMC
hsa-mir-18a	dbDEMC	hsa-mir-30a	dbDEMC
hsa-mir-146b	dbDEMC	hsa-mir-23b	dbDEMC
hsa-let-7e	dbDEMC	hsa-mir-221	dbDEMC
hsa-mir-151a	unconfirmed	hsa-mir-625	dbDEMC
hsa-mir-29b	dbDEMC	hsa-mir-122	Unconfirmed
hsa-mir-181a	dbDEMC	hsa-mir-95	dbDEMC
hsa-let-7i	dbDEMC	hsa-mir-424	dbDEMC
hsa-mir-224	dbDEMC	hsa-mir-30d	dbDEMC

Lymphoma is a group of blood cell tumors that develop from lymphocytes (a type of white blood cell). Hodgkin lymphoma (HL) and non-Hodgkin lymphoma (NHL) are the two main types of lymphoma [77]. Recent experimental studies showed that hsa-mir-223 (1st in the prediction list) regulates cell growth and targets proto-oncogenes in mycosis fungoides/cutaneous T-cell lymphoma [78]. Besides, it also has been verified that plasma hsa-mir-155, hsa-mir-203, and hsa-mir-205 (2nd in the prediction list) are biomarkers for monitoring of primary cutaneous T-cell lymphomas (TCTL) [79]. Moreover, the study of Yang *et al*. suggested that hsa-mir-10b (3rd in the prediction list) contributes to osteoblast differentiation through targeting B cell lymphoma 6 (Bcl6) which provides a novel insight into understanding the molecular mechanism underlying osteoblast differentiation and suggests a potential target for inhibiting bone loss [80]. Taking lymphoma as the investigated disease and implementing MDHGI for potential miRNA-lymphoma association prediction, nine out of the top 10, 15 out of the top 20 and 44 out of the top 50 potential lymphoma-associated miRNAs were manually verified in database dbDEMC and miR2disease (See Table 3). Furthermore, in the same way as the validation of esophageal cancer, 49, 48 and 46 out of the top 50 predictions were respectively confirmed by at least one, two and three experimental literatures in PubMed (See S2 Table).

**Table 3:** Prediction of the top 50 predicted miRNAs associated with lymphoma based on known associations in HMDD V2.0 database. The prediction result was examined in dbDEMC and miR2Disease. The first column records top 1–25 related miRNAs. The third column records the top 26–50 related miRNAs.

miRNA	Evidence	miRNA	Evidence
hsa-mir-223	dbDEMC	hsa-mir-182	dbDEMC
hsa-mir-205	dbDEMC	hsa-mir-129	dbDEMC
hsa-mir-10b	dbDEMC	hsa-mir-10a	dbDEMC;miR2Disease
hsa-mir-9	dbDEMC	hsa-mir-106b	dbDEMC
hsa-mir-145	dbDEMC;miR2Disease	hsa-mir-30a	dbDEMC
hsa-mir-141	dbDEMC	hsa-mir-1	dbDEMC
hsa-mir-143	dbDEMC;miR2Disease	hsa-mir-192	dbDEMC
hsa-mir-451a	unconfirmed	hsa-mir-22	dbDEMC
hsa-mir-196a	dbDEMC	hsa-mir-199b	dbDEMC
hsa-mir-27a	dbDEMC	hsa-mir-183	dbDEMC
hsa-mir-106a	dbDEMC;miR2Disease	hsa-mir-497	dbDEMC
hsa-mir-142	unconfirmed	hsa-mir-99a	dbDEMC;miR2Disease
hsa-mir-34c	unconfirmed	hsa-mir-199a	dbDEMC
hsa-mir-34a	dbDEMC	hsa-mir-127	dbDEMC;miR2Disease
hsa-mir-31	dbDEMC	hsa-mir-27b	dbDEMC
hsa-mir-195	dbDEMC	hsa-mir-193a	unconfirmed
hsa-mir-181b	dbDEMC	hsa-mir-148a	dbDEMC
hsa-mir-34b	dbDEMC	hsa-mir-130a	dbDEMC
hsa-mir-125b	unconfirmed	hsa-mir-224	dbDEMC
hsa-mir-429	unconfirmed	hsa-let-7a	dbDEMC
hsa-mir-7	dbDEMC	hsa-mir-197	dbDEMC
hsa-mir-214	dbDEMC	hsa-mir-137	dbDEMC
hsa-mir-29a	dbDEMC	hsa-mir-30d	dbDEMC
hsa-mir-25	dbDEMC	hsa-mir-134	dbDEMC
hsa-mir-93	dbDEMC	hsa-mir-296	dbDEMC

To facilitate further validation and research, we have provided the complete prediction list of potential miRNAs associated with all the 383 human diseases in HMDD V2.0, together with the association scores predicted by MDHGI (See S3 Table).

In addition, to illustrate the applicability of MDHGI to new diseases, namely, diseases that have no known associated miRNAs, we carried out another case study on Lung Neoplasms. Known associations for this disease were removed from the training dataset, so that predictions would only be made from the information of other diseases’ related miRNAs and the similarity measures. After implementing MDHGI, we obtained the ranking of Lung Neoplasms’ candidate miRNAs in terms of their association scores (See Table 4). The data provided by Babu *et al*. suggested that increased expression of hsa-mir-20a (1st in the prediction list) in lung cancer may decrease iron export which will lead to intracellular iron retention and cell proliferation [81]. Besides, recent research showed that hsa-mir-17 (2nd in the prediction list) and hsa-mir-92 families play important roles in cisplatin resistance and can be used as potential biomarkers for better predicting the clinical response to platinum-based chemotherapy in non-small cell lung cancer (NSCLC) [82]. Shen *et al*. provided the evidence that down-regulation of hsa-mir-18a (3rd in the prediction list) sensitizes NSCLC to radiation treatment and it may help to develop a new approach to sensitizing radioresistant lung cancer cells by targeting hsa-mir-18a [83]. Respectively, 10, 20 and 50 out of the top 10, 20 and 50 predictions were manually confirmed in HMDD V2.0, dbDEMC and miR2Disease. Similarly, we also manually verified the top 50 predicted miRNAs in PubMed. The result showed that 50 out of the top 50 predictions were confirmed by at least three experimental literatures in PubMed (See S4 Table).

**Table 4:** Prediction of the top 50 predicted miRNAs associated with lung neoplasms based on known associations in HMDD V2.0 database. All known associations between the miRNAs and Lung Neoplasms were removed before the prediction process. The prediction result was examined in dbDEMC, miR2Disease and HMDD V2.0. The first column records top 1–25 related miRNAs. The third column records the top 26–50 related miRNAs.

miRNA	Evidence	miRNA	Evidence
hsa-mir-20a	dbDEMC;miR2Disease;HMDD	hsa-mir-34c	dbDEMC;HMDD
hsa-mir-17	miR2Disease;HMDD	hsa-mir-200a	dbDEMC;miR2Disease;HMDD
hsa-mir-18a	dbDEMC;miR2Disease;HMDD	hsa-mir-146a	dbDEMC;miR2Disease;HMDD
hsa-mir-19b	dbDEMC;HMDD	hsa-mir-223	HMDD
hsa-mir-19a	dbDEMC;miR2Disease;HMDD	hsa-mir-143	dbDEMC;miR2Disease;HMDD
hsa-mir-145	dbDEMC;miR2Disease;HMDD	hsa-mir-29a	dbDEMC;miR2Disease;HMDD
hsa-mir-155	dbDEMC;miR2Disease;HMDD	hsa-let-7g	dbDEMC;miR2Disease;HMDD
hsa-let-7a	dbDEMC;miR2Disease;HMDD	hsa-mir-146b	miR2Disease;HMDD
hsa-mir-21	dbDEMC;miR2Disease;HMDD	hsa-mir-9	miR2Disease;HMDD
hsa-mir-34a	dbDEMC;HMDD	hsa-mir-218	dbDEMC;miR2Disease;HMDD
hsa-let-7b	miR2Disease;HMDD	hsa-mir-141	dbDEMC;miR2Disease
hsa-mir-92a	HMDD	hsa-mir-200c	dbDEMC;miR2Disease;HMDD
hsa-mir-126	dbDEMC;miR2Disease;HMDD	hsa-mir-106b	dbDEMC
hsa-let-7d	dbDEMC;miR2Disease;HMDD	hsa-mir-34b	dbDEMC;HMDD
hsa-let-7c	dbDEMC;miR2Disease;HMDD	hsa-mir-101	dbDEMC;miR2Disease;HMDD
hsa-mir-200b	dbDEMC;miR2Disease;HMDD	hsa-mir-15a	dbDEMC
hsa-mir-221	dbDEMC;HMDD	hsa-mir-214	dbDEMC;miR2Disease;HMDD
hsa-let-7e	miR2Disease;HMDD	hsa-mir-205	dbDEMC;miR2Disease;HMDD
hsa-mir-125b	miR2Disease;HMDD	hsa-mir-1	dbDEMC;miR2Disease;HMDD
hsa-let-7f	miR2Disease;HMDD	hsa-mir-125a	dbDEMC;miR2Disease;HMDD
hsa-mir-199a	dbDEMC;miR2Disease;HMDD	hsa-mir-10b	dbDEMC;HMDD
hsa-mir-16	dbDEMC;miR2Disease	hsa-mir-25	dbDEMC;HMDD
hsa-let-7i	dbDEMC;HMDD	hsa-mir-181b	dbDEMC;HMDD
hsa-mir-29b	dbDEMC;miR2Disease;HMDD	hsa-mir-210	dbDEMC;miR2Disease;HMDD
hsa-mir-222	dbDEMC;HMDD	hsa-mir-93	dbDEMC;miR2Disease;HMDD

Finally, we trained our model with the dataset from the HMDD V1.0 to demonstrate that MDHGI would perform equally well on different datasets. Breast Neoplasms was used as the investigated disease. As a result, there were respectively 10, 20, and 48 out of the top 10, 20 and 50 predictions manually confirmed in the three databases mentioned above (See Table 5). Besides, 50 out of the top 50 predictions were confirmed by at least three experimental literatures in PubMed (See S5 Table). Taking first-ranked hsa-let-7e as an example, research confirmed that umonji/Arid1 B (JARID1B) promoted breast tumor cell cycle progression through epigenetic repression of hsa-let-7e [84]. Recent experimental studies showed that breast cancer patients with low hsa-let-7b (2nd in the prediction list) expression had poor prognoses which indicated that hsa-let-7b might act as cancer suppressor gene in breast cancer development and progression by inhibiting the expression of BSG [85]. Moreover, the results of Sun *et al*. suggested that hsa-mir-223 (3rd in the prediction list) increases the sensitivity of triple-negative breast cancer stem cells (TNBCSCs) to TRAIL (tumor necrosis factor-related apoptosis-inducing ligand)-induced apoptosis by targeting HCLS1 (hematopoietic cell-specific Lyn substrate 1)-associated protein X-1 (HAX-1) [86].

**Table 5:** Prediction of the top 50 predicted miRNAs associated with breast neoplasms based on known associations in HMDD V1.0 database. The prediction result was examined in dbDEMC, miR2Disease and HMDD V2.0. The first column records top 1–25 related miRNAs. The third column records the top 26–50 related miRNAs.

miRNA	Evidence	miRNA	Evidence
hsa-let-7e	dbDEMC;HMDD	hsa-mir-23b	dbDEMC;HMDD
hsa-let-7b	dbDEMC;HMDD	hsa-mir-203	dbDEMC;miR2Disease; HMDD
hsa-mir-223	dbDEMC;HMDD	hsa-mir-30e	Unconfirmed
hsa-mir-126	dbDEMC;miR2Disease; HMDD	hsa-mir-29c	dbDEMC;miR2Disease; HMDD
hsa-mir-16	dbDEMC;HMDD	hsa-mir-107	dbDEMC;HMDD
hsa-let-7i	dbDEMC;miR2Disease; HMDD	hsa-mir-199b	dbDEMC;HMDD
hsa-let-7c	dbDEMC;HMDD	hsa-mir-18b	dbDEMC;HMDD
hsa-mir-92b	dbDEMC	hsa-mir-181a	dbDEMC;miR2Disease; HMDD
hsa-mir-99b	dbDEMC	hsa-mir-532	dbDEMC
hsa-mir-100	dbDEMC;HMDD	hsa-mir-27a	dbDEMC;miR2Disease; HMDD
hsa-mir-130a	dbDEMC	hsa-mir-22	dbDEMC;miR2Disease; HMDD
hsa-mir-182	dbDEMC;miR2Disease; HMDD	hsa-mir-148a	dbDEMC;miR2Disease; HMDD
hsa-mir-92a	HMDD	hsa-mir-192	dbDEMC
hsa-let-7g	dbDEMC;HMDD	hsa-mir-196b	dbDEMC
hsa-mir-106a	dbDEMC	hsa-mir-142	Unconfirmed
hsa-mir-335	dbDEMC;miR2Disease; HMDD	hsa-mir-372	dbDEMC
hsa-mir-195	dbDEMC;miR2Disease; HMDD	hsa-mir-135a	dbDEMC;HMDD
hsa-mir-150	dbDEMC	hsa-mir-224	dbDEMC;HMDD
hsa-mir-101	dbDEMC;miR2Disease; HMDD	hsa-mir-424	dbDEMC
hsa-mir-191	dbDEMC;miR2Disease; HMDD	hsa-mir-198	dbDEMC
hsa-mir-24	dbDEMC;HMDD	hsa-mir-28	dbDEMC
hsa-mir-99a	dbDEMC	hsa-mir-212	dbDEMC
hsa-mir-30a	miR2Disease;HMDD	hsa-mir-497	dbDEMC;miR2Disease; HMDD
hsa-mir-32	dbDEMC	hsa-mir-520c	miR2Disease;HMDD
hsa-mir-373	dbDEMC;miR2Disease; HMDD	hsa-mir-520b	dbDEMC;HMDD

According to the results presented, MDHGI consistently achieved an excellent predictive performance in each of the four case studies. With the continuous experimental research on miRNA-disease associations, we expect that more and more miRNAs in the prediction lists generated by our model would be verified in the future.

## Discussion

This paper introduced the computational method called MDHGI in which we combined the sparse learning method with the heterogeneous graph inference method to calculate potential miRNA-disease association scores. In the process of low-rank matrix decomposition, the sparse norm could effectively handle training datasets with a high level of noises and a low quality, which were commonly faced by biological researchers. However, some elements with the value of 1 in the adjacency matrix might turn into 0 after using the sparse learning method, which means the corresponding known miRNA-disease associations information might be removed. To overcome the disadvantage, the heterogeneous graph inference method was used by integrating the Gaussian interaction profile kernel similarity, the disease semantic similarity, the miRNA functional similarity, and miRNA-disease associations which were reacquired from the recalculated adjacency matrix into a heterogeneous graph. The excellent performance of MDHGI was demonstrated by experimental results from both cross validation and case studies on Esophageal Neoplasms, Lymphoma, Lung Neoplasms and Breast Neoplasms. It could be concluded that MDHGI should serve as an effective tool for predicting potential miRNA-disease associations, and would be helpful in human disease prevention, treatment, diagnosis, and prognosis.

The reliable performance of MDHGI came from the following factors. Firstly, by decomposing the original data into a clean (a linear combination of low-rank matrix and the adjacency matrix) and noise (sparse matrix) parts, we could obtain a clean data about the associations between miRNAs and diseases. Secondly, more and more disease-miRNA association data had been discovered and confirmed. Due to the data-dependent property of sparse learning method, the increasing number of known associations improved the prediction accuracy. Thirdly, MDHGI could be used to make predictions for new diseases which have no known related miRNAs and miRNAs without any known associated diseases. Lastly, MDHGI could effectively uncover missing miRNA-disease associations for all diseases simultaneously. Therefore, MDHGI is a superior model over previous ones.

Limitations also exist in this method. Firstly, though current studies benefited from the increased known data, it is never a finished work to expand data. Secondly, it is obvious that assigning different penalization parameters for the three different types of edges (miRNA-miRNA edges, disease-disease edges and miRNA-disease edges) would be more accurate for the prediction performance. However, there are some difficulties that make us unable to do this work. Firstly, for the moment, we don't know how to properly give different weights to vertexes and edges in the network. Secondly, since all the known miRNA-disease associations we utilized in our model were based on databases (i.e., different experimental literatures), it is very difficult for us to quantify the reliability of different edges. Hence, taking full account of your suggestions, we will conduct our research in this area in the next step. In addition, the parameter set in the algorithm is difficult to optimize, and deserves further research. Finally, MDHGI might cause bias to miRNAs which have more associated disease records and vice versa. Therefore, we would develop optimization strategies to improve the accuracy of this prediction method in the future.

## Supporting information

S1 Text The specific proof process of Theorem 1. (DOCX)

S1 Table Prediction of the top 50 predicted miRNAs associated with Esophageal Neoplasms based on known associations in HMDD V2.0 database. The prediction result was examined by experimental literatures in PubMed. The first column records top 1–25 related miRNAs. The third column records the top 26–50 related miRNAs.
(XLSX)

S2 Table Prediction of the top 50 predicted miRNAs associated with lymphoma based on known associations in HMDD V2.0 database. The prediction result was examined by experimental literatures in PubMed. The first column records top 1–25 related miRNAs. The third column records the top 26–50 related miRNAs.
(XLSX)

S3 Table We applied MDHGI to prioritize all the candidate miRNA-disease pairs based on all the known miRNA-disease associations in HMDD V2.0 database as training samples. This prediction result is released for further experimental validation and research.
(XLSX)

S4 Table Prediction of the top 50 predicted miRNAs associated with lung neoplasms based on known associations in HMDD V2.0 database. The prediction result was examined by experimental literatures in PubMed. The first column records top 1–25 related miRNAs. The third column records the top 26–50 related miRNAs.
(XLSX)

S5 Table Prediction of the top 50 predicted miRNAs associated with breast neoplasms based on known associations in HMDD V1.0 database. The prediction result was examined by experimental literatures in PubMed. The first column records top 1–25 related miRNAs. The third column records the top 26–50 related miRNAs.
(XLSX)

## References

[1] Ambros V (2004) The functions of animal microRNAs. Nature 431: 350–355. 10.1038/nature02871 15372042

[2] Bartel DP (2004) MicroRNAs: genomics, biogenesis, mechanism, and function. Cell 116: 281–297. 14744438 10.1016/s0092-8674(04)00045-5

[3] Meister G, Tuschl T (2004) Mechanisms of gene silencing by double-stranded RNA. Nature 431: 343–349. 10.1038/nature02873 15372041

[4] Ambros V (2001) microRNAs: tiny regulators with great potential. Cell 107: 823–826. 11779458 10.1016/s0092-8674(01)00616-x

[5] Lee RC, Feinbaum RL, Ambros V (1993) The C. elegans heterochronic gene lin-4 encodes small RNAs with antisense complementarity to lin-14. Cell 75: 843–854. 8252621 10.1016/0092-8674(93)90529-y

[6] Wightman B, Ha I, Ruvkun G (1993) Posttranscriptional regulation of the heterochronic gene lin-14 by lin-4 mediates temporal pattern formation in C. elegans. Cell 75: 855–862. 8252622 10.1016/0092-8674(93)90530-4

[7] Kozomara A, Griffiths-Jones S (2014) miRBase: annotating high confidence microRNAs using deep sequencing data. Nucleic acids research 42: D68–73. 10.1093/nar/gkt1181 24275495 PMC3965103

[8] Xu PZ, Guo M, Hay BA (2004) MicroRNAs and the regulation of cell death. Trends Genet 20: 617–624. 10.1016/j.tig.2004.09.010 15522457

[9] Bartel DP (2009) MicroRNAs: Target Recognition and Regulatory Functions. Cell 136: 215–233. 10.1016/j.cell.2009.01.002 19167326 PMC3794896

[10] Karp X, Ambros V (2005) Encountering microRNAs in cell fate signaling. Science 310: 1288–1289. 10.1126/science.1121566 16311325

[11] Miska EA (2005) How microRNAs control cell division, differentiation and death. Curr Opin Genet Dev 15: 563–568. 10.1016/j.gde.2005.08.005 16099643

[12] Cheng AM, Byrom MW, Shelton J, Ford LP (2005) Antisense inhibition of human miRNAs and indications for an involvement of miRNA in cell growth and apoptosis. Nucleic Acids Research 33: 1290–1297. 10.1093/nar/gki200 15741182 PMC552951

[13] Lee RC, Feinbaum RL, Ambros V (1993) The C. elegans heterochronic gene lin-4 encodes small RNAs with antisense complementarity to lin-14. Cell 89: 1828–1835. 10.1016/0092-8674(93)90529-y 8252621

[14] Esquela-Kerscher A, Slack FJ (2006) Oncomirs—microRNAs with a role in cancer. Nature reviews Cancer 6: 259–269. 10.1038/nrc1840 16557279

[15] Bommer GT, Gerin I, Feng Y, Kaczorowski AJ, Kuick R, et al (2007) p53-mediated activation of miRNA34 candidate tumor-suppressor genes. Current biology: CB 17: 1298–1307. 10.1016/j.cub.2007.06.068 17656095

[16] Latronico MV, Catalucci D, Condorelli G (2007) Emerging role of microRNAs in cardiovascular biology. Circulation research 101: 1225–1236. 10.1161/CIRCRESAHA.107.163147 18063818

[17] Lynam-Lennon N, Maher SG, Reynolds JV (2009) The roles of microRNA in cancer and apoptosis. Biol Rev 84: 55–71. 10.1111/j.1469-185X.2008.00061.x 19046400

[18] Meola N, Gennarino VA, Banfi S (2009) microRNAs and genetic diseases. PathoGenetics 2: 1–14. 10.1186/1755-8417-2-1 19889204 PMC2778645

[19] Calin GA, Croce CM (2006) MicroRNA signatures in human cancers. Nature reviews Cancer 6: 857–866. 10.1038/nrc1997 17060945

[20] Sun Y, Tawara I, Zhao M, Qin ZS, Toubai T, et al (2013) Allogeneic T cell responses are regulated by a specific miRNA-mRNA network. The Journal of clinical investigation 123: 4739–4754. 10.1172/JCI70013 24216511 PMC3809794

[21] Yuan Y, Ren X, Xie Z, Wang X (2016) A quantitative understanding of microRNA-mediated competing endogenous RNA regulation. Quantitative Biology 4: 47–57.

[22] Chen J, Zhu D, Sun Y (2017) Cap-seq reveals complicated miRNA transcriptional mechanisms in C. elegans and mouse. Quantitative Biology 5: 352–367.

[23] Song T, Zhang X, Zhang L, Dong J, Cai W, et al (2013) miR-708 promotes the development of bladder carcinoma via direct repression of Caspase-2. Journal of cancer research and clinical oncology 139: 1189–1198. 10.1007/s00432-013-1392-6 23568547 PMC11824749

[24] Pandi G, Nakka VP, Dharap A, Roopra A, Vemuganti R (2013) MicroRNA miR-29c down-regulation leading to de-repression of its target DNA methyltransferase 3a promotes ischemic brain damage. PloS one 8: e58039 10.1371/journal.pone.0058039 23516428 PMC3596316

[25] Peng CH, Liu JH, Woung LC, Lin TJ, Chiou SH, et al (2012) MicroRNAs and cataracts: correlation among let-7 expression, age and the severity of lens opacity. The British journal of ophthalmology 96: 747–751. 10.1136/bjophthalmol-2011-300585 22334139

[26] Han K, Xuan P, Ding J, Zhao ZJ, Hui L, et al (2014) Prediction of disease-related microRNAs by incorporating functional similarity and common association information. Genetics and molecular research: GMR 13: 2009–2019. 10.4238/2014.March.24.5 24737426

[27] You ZH, Huang ZA, Zhu Z, Yan GY, Li ZW, et al (2017) PBMDA: A novel and effective path-based computational model for miRNA-disease association prediction. PLoS computational biology 13: e1005455 10.1371/journal.pcbi.1005455 28339468 PMC5384769

[28] Chen X, Gong Y, Zhang DH, You ZH, Li ZW (2017) DRMDA: deep representations-based miRNA-disease association prediction. Journal of cellular and molecular medicine 10.1111/jcmm.13336 28857494 PMC5742725

[29] Chen X, Niu YW, Wang GH, Yan GY (2017) HAMDA: Hybrid Approach for MiRNA-Disease Association prediction. Journal of biomedical informatics 10.1016/j.jbi.2017.10.014 29097278

[30] Peng L, Chen Y, Ma N, Chen X (2017) NARRMDA: negative-aware and rating-based recommendation algorithm for miRNA-disease association prediction. Molecular bioSystems 10.1039/c7mb00499k 29053164

[31] You ZH, Wang LP, Chen X, Zhang S, Li XF, et al (2017) PRMDA: personalized recommendation-based MiRNA-disease association prediction. Oncotarget. 10.18632/oncotarget.20996 PMC5689632 29156742

[32] Chen X, Jiang ZC, Xie D, Huang DS, Zhao Q, et al (2017) A novel computational model based on super-disease and miRNA for potential miRNA-disease association prediction. Molecular bioSystems 13: 1202–1212. 10.1039/c6mb00853d 28470244

[33] Chen X, Xie D, Zhao Q, You ZH (2017) MicroRNAs and complex diseases: from experimental results to computational models. Briefings in bioinformatics 10.1093/bib/bbx130 29045685

[34] Jiang Q, Hao Y, Wang G, Juan L, Zhang T, et al (2010) Prioritization of disease microRNAs through a human phenome-microRNAome network. BMC systems biology 4 Suppl 1: S2. 10.1186/1752-0509-4-S1-S2 PMC2880408 20522252

[35] Shi H, Xu J, Zhang G, Xu L, Li C, et al (2013) Walking the interactome to identify human miRNA-disease associations through the functional link between miRNA targets and disease genes. BMC systems biology 7: 101 10.1186/1752-0509-7-101 24103777 PMC4124764

[36] Mork S, Pletscher-Frankild S, Palleja Caro A, Gorodkin J, Jensen LJ (2014) Protein-driven inference of miRNA-disease associations. Bioinformatics (Oxford, England) 30: 392–397. 10.1093/bioinformatics/btt677 PMC3904518 24273243

[37] Xuan P, Han K, Guo M, Guo Y, Li J, et al (2013) Correction: Prediction of microRNAs Associated with Human Diseases Based on Weighted k Most Similar Neighbors. PloS one 8. 10.1371/journal.pone.0070204 PMC3738541 23950912

[38] Chen X, Liu MX, Yan GY (2012) RWRMDA: predicting novel human microRNA-disease associations. Molecular bioSystems 8: 2792–2798. 10.1039/c2mb25180a 22875290

[39] Xuan P, Han K, Guo Y, Li J, Li X, et al (2015) Prediction of potential disease-associated microRNAs based on random walk. Bioinformatics (Oxford, England) 31: 1805–1815. 10.1093/bioinformatics/btv039 25618864

[40] Li JQ, Rong ZH, Chen X, Yan GY, You ZH (2017) MCMDA: Matrix completion for MiRNA-disease association prediction. Oncotarget 8: 21187–21199. doi: 10.18632/oncotarget.15061 28177900 10.18632/oncotarget.15061 PMC5400576

[41] Chen X, Yan CC, Zhang X, You ZH, Deng L, et al (2016) WBSMDA: Within and Between Score for MiRNA-Disease Association prediction. Scientific reports 6: 21106 10.1038/srep21106 26880032 PMC4754743

[42] Chen X, Yan CC, Zhang X, You ZH, Huang YA, et al (2016) HGIMDA: Heterogeneous graph inference for miRNA-disease association prediction. Oncotarget 7: 65257–65269. doi: 10.18632/oncotarget.11251 27533456 10.18632/oncotarget.11251 PMC5323153

[43] Xu J, Li CX, Lv JY, Li YS, Xiao Y, et al (2011) Prioritizing candidate disease miRNAs by topological features in the miRNA target-dysregulated network: case study of prostate cancer. Molecular cancer therapeutics 10: 1857–1866. 10.1158/1535-7163.MCT-11-0055 21768329

[44] Chen X, Yan GY (2014) Semi-supervised learning for potential human microRNA-disease associations inference. Scientific reports 4: 5501 10.1038/srep05501 24975600 PMC4074792

[45] Chen X, Yan CC, Zhang X, Li Z, Deng L, et al (2015) RBMMMDA: predicting multiple types of disease-microRNA associations. Scientific reports 5: 13877 10.1038/srep13877 26347258 PMC4561957

[46] Chen X, Wu QF, Yan GY (2017) RKNNMDA: Ranking-based KNN for MiRNA-Disease Association prediction. RNA biology 14: 952–962. 10.1080/15476286.2017.1312226 28421868 PMC5546566

[47] Zhong Y, Xuan P, Wang X, Zhang T, Li J, et al (2017) A non-negative matrix factorization based method for predicting disease-associated miRNAs in miRNA-disease bilayer network. Bioinformatics (Oxford, England) 10.1093/bioinformatics/btx546 28968753 PMC5860200

[48] Xiao Q, Luo J, Liang C, Cai J, Ding P (2017) A graph regularized non-negative matrix factorization method for identifying microRNA-disease associations. Bioinformatics (Oxford, England) 10.1093/bioinformatics/btx545 28968779

[49] Zeng X, Liu L, Lu L, Zou Q (2018) Prediction of potential disease-associated microRNAs using structural perturbation method. Bioinformatics (Oxford, England) 10.1093/bioinformatics/bty112 29490018

[50] Chen X, Huang YA, Wang XS, You ZH, Chan KC (2016) FMLNCSIM: fuzzy measure-based lncRNA functional similarity calculation model. Oncotarget 7: 45948–45958. doi: 10.18632/oncotarget.10008 27322210 10.18632/oncotarget.10008 PMC5216773

[51] Huang YA, Chen X, You ZH, Huang DS, Chan KCC (2016) ILNCSIM: improved lncRNA functional similarity calculation model. Oncotarget 7: 25902–25914. doi: 10.18632/oncotarget.8296 27028993 10.18632/oncotarget.8296 PMC5041953

[52] Chen X, Yan CC, Zhang X, You ZH (2016) Long non-coding RNAs and complex diseases: from experimental results to computational models. Briefings in Bioinformatics 10.1093/bib/bbw060 27345524 PMC5862301

[53] Chen X (2015) KATZLDA: KATZ measure for the lncRNA-disease association prediction. Scientific reports 5: 16840 10.1038/srep16840 26577439 PMC4649494

[54] Chen X, Yan CC, Luo C, Ji W, Zhang Y, et al (2015) Constructing lncRNA functional similarity network based on lncRNA-disease associations and disease semantic similarity. Scientific reports 5: 11338 10.1038/srep11338 26061969 PMC4462156

[55] Wright J, Ganesh A, Rao S, Ma Y (2009) Robust Principal Component Analysis: Exact Recovery of Corrupted Low-Rank Matrices. Advances in Neural Information Processing Systems 87: 20:23–20:56.

[56] Wang D, Wang JA, Lu M, Song F, Cui QH (2010) Inferring the human microRNA functional similarity and functional network based on microRNA-associated diseases. Bioinformatics 26: 1644–1650. 10.1093/bioinformatics/btq241 20439255

[57] Van LT, Nabuurs SB, Marchiori E (2011) Gaussian interaction profile kernels for predicting drug-target interaction. Bioinformatics 27: 3036–3043. 10.1093/bioinformatics/btr500 21893517

[58] Chen X, Huang YA, You ZH, Yan GY, Wang XS (2016) A novel approach based on KATZ measure to predict associations of human microbiota with non-infectious diseases. Bioinformatics 10.1093/bioinformatics/btw715: btw715 28025197

[59] Chen X, Yan GY (2013) Novel human lncRNA-disease association inference based on lncRNA expression profiles. Bioinformatics (Oxford, England) 29: 2617–2624. 10.1093/bioinformatics/btt426 24002109

[60] Pech R, Hao D, Po M, Zhou T (2017) Predicting drug-target interactions via sparse learning.

[61] Liu G, Lin Z, Yan S, Sun J, Yu Y, et al (2013) Robust recovery of subspace structures by low-rank representation. IEEE transactions on pattern analysis and machine intelligence 35: 171–184. 10.1109/TPAMI.2012.88 22487984

[62] Chen J, Yang J (2014) Robust subspace segmentation via low-rank representation. IEEE transactions on cybernetics 44: 1432–1445. 10.1109/TCYB.2013.2286106 24196982

[63] Chandrasekaran V, Sanghavi S, Parrilo PA, Willsky AS (2009) Rank-Sparsity Incoherence for Matrix Decomposition. Siam Journal on Optimization 21: 572–596.

[64] Meng F, Yang X, Zhou C (2014) The augmented lagrange multipliers method for matrix completion from corrupted samplings with application to mixed Gaussian-impulse noise removal. PloS one 9: e108125 10.1371/journal.pone.0108125 25248103 PMC4172684

[65] Wang W, Yang S, Li J (2013) Drug target predictions based on heterogeneous graph inference. Pacific Symposium on Biocomputing Pacific Symposium on Biocomputing: 53–64. 23424111 PMC3605000

[66] Vanunu O, Magger O, Ruppin E, Shlomi T, Sharan R (2010) Associating genes and protein complexes with disease via network propagation. PLoS computational biology 6: e1000641 10.1371/journal.pcbi.1000641 20090828 PMC2797085

[67] Yu H, Chen X, Lu L (2017) Large-scale prediction of microRNA-disease associations by combinatorial prioritization algorithm. Scientific reports 7: 43792 10.1038/srep43792 28317855 PMC5357838

[68] Xuan P, Han K, Guo M, Guo Y, Li J, et al (2013) Prediction of microRNAs associated with human diseases based on weighted k most similar neighbors. PloS one 8: e70204 10.1371/journal.pone.0070204 23950912 PMC3738541

[69] Pasquier C, Gardes J (2016) Prediction of miRNA-disease associations with a vector space model. Scientific reports 6: 27036. 10.1038/srep27036 PMC4887905 27246786

[70] Li Y, Qiu C, Tu J, Geng B, Yang J, et al (2014) HMDD v2.0: a database for experimentally supported human microRNA and disease associations. Nucleic acids research 42: D1070–1074. 10.1093/nar/gkt1023 24194601 PMC3964961

[71] Yang Z, Ren F, Liu C, He S, Sun G, et al (2010) dbDEMC: a database of differentially expressed miRNAs in human cancers. BMC genomics 11 Suppl 4: S5. 10.1186/1471-2164-11-S4-S5 PMC3005935 21143814

[72] Jiang Q, Wang Y, Hao Y, Juan L, Teng M, et al (2009) miR2Disease: a manually curated database for microRNA deregulation in human disease. Nucleic acids research 37: D98–104. 10.1093/nar/gkn714 18927107 PMC2686559

[73] American Cancer Society: Cancer Facts and Figures 2017 Atlanta, Ga: American Cancer Society, 2017.

[74] Zhang HF, Zhang K, Liao LD, Li LY, Du ZP, et al (2014) miR-200b suppresses invasiveness and modulates the cytoskeletal and adhesive machinery in esophageal squamous cell carcinoma cells via targeting Kindlin-2. Carcinogenesis 35: 292–301. 10.1093/carcin/bgt320 24064224

[75] Chen G, Peng J, Zhu W, Tao G, Song Y, et al (2014) Combined downregulation of microRNA-133a and microRNA-133b predicts chemosensitivity of patients with esophageal squamous cell carcinoma undergoing paclitaxel-based chemotherapy. Medical oncology (Northwood, London, England) 31: 263. 10.1007/s12032-014-0263-6 25280517

[76] Zhu Y, Xia Y, Niu H, Chen Y (2014) MiR-16 induced the suppression of cell apoptosis while promote proliferation in esophageal squamous cell carcinoma. Cellular physiology and biochemistry: international journal of experimental cellular physiology, biochemistry, and pharmacology 33: 1340–1348. 10.1159/000358701 24852767

[77] Alizadeh AA, Eisen MB, Davis RE, Ma C, Lossos IS, et al (2000) Distinct types of diffuse large B-cell lymphoma identified by gene expression profiling. Nature 403: 503–511. 10.1038/35000501 10676951

[78] McGirt LY, Adams CM, Baerenwald DA, Zwerner JP, Zic JA, et al (2014) miR-223 regulates cell growth and targets proto-oncogenes in mycosis fungoides/cutaneous T-cell lymphoma. The Journal of investigative dermatology 134: 1101–1107. 10.1038/jid.2013.461 24304814 PMC3961555

[79] Dusilkova N, Basova P, Polivka J, Kodet O, Kulvait V, et al (2017) Plasma miR-155, miR-203, and miR-205 are Biomarkers for Monitoring of Primary Cutaneous T-Cell Lymphomas. International journal of molecular sciences 18. 10.3390/ijms18102136 PMC5666818 29036928

[80] Yang J, Wang S, Wang F, Mu X, Qu Y, et al (2017) Downregulation of miR-10b promotes osteoblast differentiation through targeting Bcl6. International journal of molecular medicine 39: 1605–1612. 10.3892/ijmm.2017.2955 28440396

[81] Babu KR, Muckenthaler MU (2016) miR-20a regulates expression of the iron exporter ferroportin in lung cancer. Journal of molecular medicine (Berlin, Germany) 94: 347–359. 10.1007/s00109-015-1362-3 PMC4803811 26560875

[82] Zhao J, Fu W, Liao H, Dai L, Jiang Z, et al (2015) The regulatory and predictive functions of miR-17 and miR-92 families on cisplatin resistance of non-small cell lung cancer. BMC cancer 15: 731 10.1186/s12885-015-1713-z 26482648 PMC4617718

[83] Shen Z, Wu X, Wang Z, Li B, Zhu X (2015) Effect of miR-18a overexpression on the radiosensitivity of non-small cell lung cancer. International journal of clinical and experimental pathology 8: 643–648. 25755757 PMC4348909

[84] Mitra D, Das PM, Huynh FC, Jones FE (2011) Jumonji/ARID1 B (JARID1B) protein promotes breast tumor cell cycle progression through epigenetic repression of microRNA let-7e. The Journal of biological chemistry 286: 40531–40535. 10.1074/jbc.M111.304865 21969366 PMC3220509

[85] Ma L, Li GZ, Wu ZS, Meng G (2014) Prognostic significance of let-7b expression in breast cancer and correlation to its target gene of BSG expression. Medical oncology (Northwood, London, England) 31: 773. 10.1007/s12032-013-0773-7 24264599

[86] Sun X, Li Y, Zheng M, Zuo W, Zheng W (2016) MicroRNA-223 Increases the Sensitivity of Triple-Negative Breast Cancer Stem Cells to TRAIL-Induced Apoptosis by Targeting HAX-1. PloS one 11: e0162754 10.1371/journal.pone.0162754 27618431 PMC5019415

