# Supplementary material for: MDHGI: Matrix Decomposition and Heterogeneous Graph Inference for miRNA-disease association prediction
Source: PLoS Comput Biol. 2018 Aug 24;14(8):e1006418. doi: 10.1371/journal.pcbi.1006418 (PMC6126877; doi:10.1371/journal.pcbi.1006418)
Supplement: S1 Text — (DOCX) [file pcbi.1006418.s001.docx]

**MDHGI: Matrix Decomposition and Heterogeneous Graph Inference for miRNA-disease association prediction** Xing Chen^1, *^, Jun Yin^1^, Jia Qu^1^, Li Huang^2^

^1^School of Information and Control Engineering, China University of Mining and Technology, Xuzhou, 221116, China

^2^ Business Analytics Centre, National University of Singapore, 119613, Singapore

*Corresponding author

**Email**: [xingchen@amss.ac.cn](mailto:xingchen@amss.ac.cn)

**Theorem 1.** When $SR$ and $SD$ are properly normalized utilizing Eq. (3) and Eq. (2) respectively, it is guaranteed that Eq. (1) will converge.

$P_{i+1}=a SR\times P_{i}\times SD+(1-a) A$ (1)

$SD(d_{i},d_{j})=\frac{SD(d_{i},d_{j})}{\sqrt{\sum_{l=1}^{nd} SD(d_{i},d_{l})}\sqrt{\sum_{l=1}^{nd} SD(d_{j},d_{l})}}$ (2)

$SR(m_{i},m_{j})=\frac{SR(m_{i},m_{j})}{\sqrt{\sum_{l=1}^{nm} SR(m_{i},m_{l})}\sqrt{\sum_{l=1}^{nm} SR(m_{j},m_{l})}}$ (3)

**Proof of Theorem 1**

In order to make the whole proof process more concise, we denote *SR*, *SD* and *P* to *A*, *B* and *X* respectively where *A*, *B* and *X* are $n\times n$, $m\times m$ and $n\times m$ matrices respectively. Besides, we denote $A_{i}$ and $A^{j}$ as the $i\text{-th}$ row of *A* and $j\text{-th}$column of *A* respectively. $a_{ij}$ is used to represent the value of $A(i,j)$*.* We use the similar way to define the matrix *B* and *X*. After that, based on Eq. (1), we can obtain:

$x_{ij}=\alpha A_{i}XB^{j}+(1-\alpha)x_{ij}^{0}$ (4)

As for $X^{1}$, we can also get

$\left[\begin{matrix} x_{1,1} \\ \text{ }\vdots\\ x_{n,1} \end{matrix}\right]=\alpha\left[\begin{matrix} a_{1,1}b_{1,1},\cdots,a_{1,n}b_{1,1},\cdots,a_{1,1}b_{m,1},\cdots,a_{1,n}b_{m,1} \\ \text{ }\vdots\\ a_{n,1}b_{1,1},\cdots,a_{n,n}b_{1,1},\cdots,a_{n,1}b_{m,1},\cdots,a_{n,n}b_{m,1} \end{matrix}\right]\left[\begin{matrix} x_{1,1} \\ \text{ }\vdots\\ x_{n,1} \\ \text{ }\vdots\\ x_{1,m} \\ \text{ }\vdots\\ x_{n,m} \end{matrix}\right]+(1-\alpha)\left[\begin{matrix} x_{1,1}^{0} \\ \text{ }\vdots\\ x_{n,1}^{0} \end{matrix}\right]$(5)

Here we use $A_{i}\times B^{j}$ to denote $\left[a_{i,1}b_{1,j}\text{ }\cdots\text{ }a_{i,n}b_{1,j}\text{ }\cdots\text{ }a_{i,1}b_{m,j}\text{ }\cdots\text{ }a_{i,n}b_{m,j}\right]$ and then Eq. (1) can be written as:

$\left[\begin{matrix} X^{1} \\ \text{ }\vdots\\ X^{m} \end{matrix}\right]=\alpha\left[\begin{matrix} A_{1}\times B^{1} \\ \text{ }\vdots\\ A_{n}\times B^{1} \\ \text{ }\vdots\\ A_{1}\times B^{m} \\ \text{ }\vdots\\ A_{n}\times B^{m} \end{matrix}\right]\left[\begin{matrix} X^{1} \\ \text{ }\vdots\\ X^{m} \end{matrix}\right]+(1-\alpha)\left[\begin{matrix} X^{10} \\ \text{ }\vdots\\ X^{m0} \end{matrix}\right]$ (6)

Let *C* denote $\left[A_{1}\times B^{1}\text{ }\cdots\text{ }A_{n}\times B^{1}\text{ }\cdots\text{ }A_{1}\times B^{n}\text{ }\cdots\text{ }A_{n}\times B^{m}\right]^{\mathrm{T}}$ and $i=sn+t$, $j=rn+\theta$, $s=sI\left\{t>0\right\}+(s-1)I\left\{t=0\right\}$, $t=tI\left\{t>0\right\}+nI\left\{t=0\right\}$,$r=rI\left\{\theta>0\right\}+(r-1)I\left\{\theta=0\right\}$, $\theta\text{=}\theta I\left\{\theta>0\right\}+nI\left\{\theta=0\right\}$, $0\leq t$, $\theta<n$.

Then we can get $c_{i,j}\text{=}a_{t,\theta}b_{r+1,s+1}$ and $c_{j,i}=a_{\theta,t}b_{s+1,r+1}$.

Through comparing the above two equations, we can find that *C* is a $n\times m$ symmetrical matrix. The Eq. (6) can be written as follows after using $X^{\text{*}}$ to represents $\left[X^{1}\text{ }\cdots\text{ }X^{n}\right]^{\mathrm{T}}$:

$X^{\text{*}}\text{=}\alpha CX^{*}+(1-\alpha)X^{*0}$ (7)

Since we wish to get a converged solution for Eq. (7), *C* can be normalized as $C^{norm}=D^{-1/2}CD^{-1/2}$, where *D* is a diagonal matrix with $d_{i,i}$ equals to the sum of the $i\text{-th}$ row of *C.* Hence, we can also get $c_{i,j}^{norm}=\frac{c_{i,j}}{\sqrt{d_{i,i}d_{j,j}}}$ and $d_{i,i}=\sum_{u=1}^{nm} c_{i,u}=\sum_{u=1}^{nm} a_{t,\theta_{u}}b_{r_{u}+1,s+1}=\sum_{p=1}^{n} a_{t,p}\sum_{q=1}^{m} b_{q,s+1}$ where $u=r_{u}n+\theta_{u}$. After incorporating the above equation into $c_{i,j}^{norm}$, we can obtain:

$c_{i,j}^{norm}=\frac{a_{t,\theta}b_{r+1,s+1}}{\sqrt{\sum_{p=1}^{n} a_{t,p}\sum_{q=1}^{m} b_{q,s+1}\sqrt{\sum_{p=1}^{n} a_{\theta,p}\sum_{q=1}^{m} b_{q,r+1}}}}=\frac{a_{t,\theta}}{\sqrt{\sum_{p=1}^{n} a_{t,p}\sum_{p=1}^{n} a_{\theta,p}}}\frac{b_{r+1,s+1}}{\sqrt{\sum_{q=1}^{m} b_{q,s+1}\sum_{q=1}^{m} b_{q,r+1}}}$ (8)

Therefore, if we normalize *A* and *B* as $a_{i,j}^{norm}=\frac{a_{i,j}}{\sqrt{\sum_{p=1}^{n} a_{i,p}\sum_{p=1}^{n} a_{j,p}}}$ and $b_{i,j}^{norm}=\frac{b_{i,j}}{\sqrt{\sum_{q=1}^{n} b_{q,i}\sum_{q=1}^{n} b_{q,i}}}$

We can get $c_{i,j}^{norm}=a_{t,\theta}^{norm}b_{r+1,s+1}^{norm}$. Thus, we can rewrite Eq. (7) as $X^{*}=\alpha C^{norm}X^{*}+(1-\alpha)X^{*0}$ with this equation.
